# Gua Sha Alleviates Radiculitis-Induced Pain Via HIF-1α–Mediated Metabolic Reprogramming Pathway in Rats

**DOI:** 10.1155/prm/9923147

**Published:** 2025-03-17

**Authors:** Haotian Ge, Shuxia Yan, Mingwan Yin, Yujie Gao, Jiayi Wang, Qing Wang, Guihua Xu, Min Yang

**Affiliations:** ^1^School of Nursing, Nanjing University of Chinese Medicine, Nanjing, Jiangsu, China; ^2^TCM Nursing Intervention Laboratory of Chronic Diseases, Nanjing University of Chinese Medicine, Nanjing, Jiangsu, China

**Keywords:** Gua Sha, HIF-1α, inflammation, metabolic reprogramming, radiculitis-induced pain

## Abstract

**Background:** Radiculitis-induced pain (RIP) results from dorsal root ganglion (DRG) sensitization due to inflammation. Hypoxia-inducible factor 1-alpha (HIF-1*α*) is linked to inflammatory responses through metabolic changes, but its role in RIP is not well understood. Gua Sha therapy has been shown to reduce inflammation and neural damage from lumbar disc herniation (LDH). This study investigates whether HIF-1α–mediated metabolic reprogramming contributes to the pain-relieving effects of Gua Sha in RIP.

**Methods:** Male SD rats were subjected to LDH surgery and divided into six groups: sham, model, sham Gua Sha, Gua Sha, Gua Sha + DMOG, and Gua Sha + YC-1. Gua Sha was applied 5 days postsurgery, every other day for three sessions per course, totaling three courses. Changes in paw withdrawal threshold (PWT) and latency (PWL) were monitored, along with blood flow in the rats' backs. Levels of IL-1β, TNF-*α*, and NF-κB were assessed in serum and DRG tissue. Pathological changes and hypoxia in DRG tissues were observed using hematoxylin–eosin staining and immunofluorescence. Western blotting and qPCR measured HIF-1α, GLUT1, PFKM, and PDK1 expression, while lactic acid and ATP levels in DRG tissue were also evaluated.

**Results:** Gua Sha increased PWT and PWL, reduced serum and DRG inflammatory factors, improved back microcirculation, alleviated DRG hypoxia, and decreased HIF-1α and related signaling factors. It also lowered lactic acid and raised ATP levels. DMOG, a HIF-1α activator, reversed these effects. HIF-1α activation did not affect serum inflammatory factors but partially improved PWT. Inhibition of HIF-1α with YC-1 did not significantly differ from Gua Sha alone.

**Conclusion:** HIF-1α–mediated metabolic reprogramming is a pathogenic mechanism in RIP. Gua Sha alleviates RIP by enhancing microcirculation, improving DRG hypoxia, inhibiting HIF-1α–mediated reprogramming, and reducing DRG sensitization and inflammation. This study provides insights into the mechanisms of Gua Sha's therapeutic effects in RIP.

## 1. Introduction

Radiculitis-induced pain (RIP) is a common symptom associated with lumbar disc herniation (LDH). When the intervertebral disc (IVD) protrudes, the nucleus pulposus or disc tissue causes mechanical compression on the adjacent dorsal root ganglion (DRG) or cauda equina, leading to inflammatory exudate and chemical irritation from the nucleus pulposus or disc, which induces radiating pain in the lower back or legs. This condition can transform into chronic pain, affecting patients' daily lives and work over the long term and imposing an economic burden [1, 2].

Inflammatory stimulation is the basis for RIP, and early studies revealed that degenerative IVDs and nucleus-derived inflammatory mediators such as IL-1β and TNF-*α* cause neuritis and sensitization, whereas mechanical compression only causes pain when the nerve is already sensitized [3]. Inflammation leads to tissue hypoxia, a process involving increased cellular metabolic demands and reduced metabolic substrates due to thrombosis, trauma, compression, and other factors [4]. In addition, recent *in vitro* studies have shown that hypoxia and acidic stimulation can also sensitize DRG neuronal cells, and the combined effect of the two is stronger than their individual effects [5]. Compression of the IVD can increase the pressure on nerve fluid, leading to ischemia and hypoxia in DRG tissue [6]. Hypoxia can further induce inflammation through the hypoxia signaling pathway [4]. These studies indicate that hypoxia is also an important factor leading to RIP and that improving hypoxia may be a potential way to alleviate RIP.

Hypoxia-inducible factor 1-alpha (HIF-1α) is an important oxygen regulatory factor and a key factor in mediating metabolic reprogramming. HIF-1α can be stably expressed in a low-oxygen environment and can activate key glycolytic enzymes, such as pyruvate dehydrogenase kinase 1 (PDK1), phosphofructokinase 1 (PFK1), and glucose transporter 1 (GLUT1), to increase glycolytic activity and help tissues adapt to the energy metabolic needs of the hypoxic environment [7, 8]. Metabolic reprogramming was first discovered by Warburg in cancer cells and is known as the Warburg effect, which refers to the phenomenon in which cancer cells tend to use glycolysis to obtain energy even when there is enough oxygen to support mitochondrial oxidative phosphorylation (OXPHOS) [9, 10]. With further research, HIF-1α has also been found in normal tissues other than tumors. Studies have shown that HIF-1α is stably expressed in human, rat, and sheep nucleus pulposus cells [11]. The HIF-1α signaling pathway is also significantly expressed in degenerated disc tissue and is involved in key pathways in disc degeneration [12, 13]. Therefore, DRG tissue in contact with the protruding IVD or nucleus tissue is susceptible to the influence of HIF-1α. At this time, the increase in glycolysis caused by HIF-1α results in the production of a large amount of lactic acid (LA), resulting in an acidic environment. The acidic environment can work together with the hypoxia factor that causes an increase in HIF-1α to form a stronger sensitization effect on the DRG. In addition, LA has been proven to increase the secretion of IL-1β by macrophages and increase the activity of NF-κB and the release of TNF-*α* [14]. At the same time, many studies have shown that the activation of OXPHOS can play an anti-inflammatory role, while the enhancement of glycolysis promotes inflammatory responses, and the accumulation of methylglyoxal, a glycolytic product in the DRG, can exacerbate radicular neuropathic pain [15–17]. These studies indicate that HIF-1α is likely to cause sensitization of DRGs through metabolic reprogramming, creating an acidic environment, triggering inflammatory responses, participating in the progression of RIP, and serving as a potential target for treatment.

Current treatments for RIP are mostly nonsurgical treatments, such as drug therapy and physical therapy. Drug therapy mainly relieves inflammation and pain symptoms through nonsteroidal anti-inflammatory drugs and analgesics, but its long-term effectiveness is not significant [18]. Despite the relatively low level of evidence, physical therapies such as heat therapy and traction therapy are more favored in the clinic because of their good accessibility, low cost, and significant clinical benefits [19]. Like physical therapy, Gua Sha is a unique nondrug therapy in traditional Chinese medicine that uses a specially designed smooth-edged scraping board to scrape the skin, causing petechiae or ecchymosis on the local surface of the body, which usually disappears completely within a week [20]. According to traditional Chinese medicine theory, Gua Sha can regulate qi and blood, dredge meridians, and detoxify them by stimulating acupoints and meridians and has unique advantages in treating acute and chronic pain and musculoskeletal diseases [21]. Gua Sha has been widely applied in clinical practice for the management of LDH. A prior systematic review, which synthesized the findings from multiple clinical studies, has indicated that Gua Sha exhibits significant clinical efficacy in alleviating pain among patients with various types of LDH [22]. However, the underlying mechanism of its action remains unclear. Previous experimental studies have shown that Gua Sha can reduce the expression of inflammatory factors such as IL-1β, IL-6, TNF-*α*, substance P (SP), and neuropeptide Y (NPY) in the serum of LDH rats and reduce the expression of phospholipase A2 (PLA2) and prostaglandin E2 (PGE2) in DRG tissue, thereby prolonging the paw withdrawal latency (PWL) of LDH rats [23, 24]. These findings indicate that Gua Sha can improve the immune inflammatory response and pain sensitivity caused by LDH and reduce neural damage. Our previous proteomic research revealed that the expression of the PFKM protein in DRG tissue decreased after Gua Sha, indicating that the mechanism of Gua Sha intervention in RIP may be related to the regulation of glycolysis. We speculate that the mechanism by which Gua Sha regulates glycolysis may be related to HIF-1α. Increasing blood flow can enhance the transport of oxygen, thereby improving local tissue hypoxia. Studies have also shown that Gua Sha promotes microcirculation perfusion in the treatment area [21]. However, whether Gua Sha can alleviate RIP by promoting microcirculation perfusion, improving DRG tissue hypoxia, inhibiting HIF-1α–mediated metabolic reprogramming to produce excessive LA, relieving DRG sensitization, and alleviating inflammatory responses still needs to be confirmed experimentally.

This study hypothesizes that Gua Sha can alleviate RIP by promoting microcirculation perfusion, improving DRG tissue ischemia and hypoxia, and then inhibiting HIF-1α–mediated metabolic reprogramming. This experiment uses a rat model of noncompressive autologous nucleus pulposus transplantation to simulate LDH, which causes contact between the nucleus tissue and the DRG. Gua Sha intervention was implemented, and the role of HIF-1α in the alleviation of RIP by Gua Sha was verified via the HIF-1α inhibitors Lificiguat (YC-1, 3-[5-hydroxymethyl-2-furyl]-1-benzylindazole) and the HIF-1α agonist dimethyloxallyl glycine (DMOG).

## 2. Materials and Methods

### 2.1. Main Reagents and Antibodies

DMOG (HY-15893) and YC-1 (HY-14927) were purchased from MedChemExpress (Shanghai, China). The Hypoxyprobe^TM^-1 Plus Kit (HP2-100 kit) was purchased from Hypoxyprobe, Inc. (MAassachusetts, United States of America). The HE dye solution set (G1120) and LA content assay kit (bc2230) were purchased from Solarbio (Beijing, China). Rat IL-1β ELISA Kit (AF2923-A), Rat TNF-*α* ELISA Kit (AF3056-A), and Rat NF-κB ELISA Kit (AF8592-A) were purchased from AiFang Biological (Hunan, China). The ATP Assay Kit (S0026B) was purchased from Beyotime Biotechnology (Beijing, China). The FastPure Cell/Tissue Total RNA Isolation Kit was purchased from Vazyme Biotech (Jiangsu, China). All-in-one RT Mix for qPCR (R123) and qPCR SYBR Green Master Mix (High ROX) (Q401-H) were purchased from ATG Biotechnology (Jiangsu, China). NCM Western Blot Stripping Buffer (WB6200) was purchased from New Cell & Molecular Biotech (Jiangsu, China). Beta-actin monoclonal antibody (66009-1-Ig), HIF-1*α* monoclonal antibody (66730-1-Ig), GLUT1 monoclonal antibody (66290-1-Ig), PFKM polyclonal antibody (55028-1-AP), PDK1 polyclonal antibody (18262-1-AP), goat anti-mouse IgG (SA00001-1), and goat anti-rabbit IgG (SA00001-2) were purchased from Proteintech (Hubei, China).

### 2.2. Animals

The experimental design was approved by the Ethics Committee of the Nanjing University of Chinese Medicine (no.202308A063) and the study was conducted in strict accordance with the guidelines provided by the same committee. 60 healthy adult male SD rats, with a body weight of 300 ± 50 g and aged 6–8 weeks, were purchased from Shanghai SLAC Laboratory Animal Co., Ltd. The animals were not genetically modified and had not been previously used in experiments. All the rats were housed at the Laboratory Animal Center of the Nanjing University of Chinese Medicine in a housing environment with a temperature of 22–26°C, a relative humidity of 40%–60%, and an unrestricted access to water and food during the experiment. After a one-week adaptive breeding period, the 60 rats were randomly divided into the Gua Sha group, sham Gua Sha group, model group, sham surgery group, Gua Sha + DMOG group, and Gua Sha + YC-1 group via the random number table method, with 10 rats per group and 5 rats per cage. This study was conducted in accordance with the ARRIVE guidelines.

### 2.3. Model Construction

The noncompressive LDH model was established in the Gua Sha group, sham Gua Sha group, model group, Gua Sha + DMOG group, and Gua Sha + YC-1 group via the autologous nucleus pulposus transplantation method. Isoflurane inhalation anesthesia was used (flow rate: 2–3 L/min; concentration: 2.5–3). Under sterile conditions, a longitudinal incision was made along the midline of the back, 15 mm above and below the iliac crest (L6), through the skin and fascia, and the right paravertebral muscles were bluntly separated to expose the L4–5 and L5–6 intervertebral joints. A right L4 and L5 lower facetectomy, L5 and L6 upper facetectomy, and L4 and L5 hemilaminectomy were performed to expose the right L4 and L5 DRGs. The rat's tail was cut approximately 2 cm from the anus, the autologous nucleus pulposus tissue (approximately 4 mg) was immediately placed on the exposed L4 and L5 DRGs, avoiding mechanical compression, and the back and tail incisions were sutured layer by layer. After the operation, erythromycin ointment was applied to the wound surface, and 40 k U/d penicillin was administered via intramuscular injection for 3 days to prevent surgical infection. The sham surgery group rats were exposed to the right L4 and L5 DRGs after the tail was removed but without placing the nucleus, and the rest of the process was the same. During the postoperative period, the rats were observed for any signs of unsteady gait or foot eversion to determine if spinal cord injury had occurred, and rats with spinal cord injury were excluded. In this study, no rats died or experienced spinal cord injury due to model construction.

### 2.4. Intervention Methods

After a 5-day recovery period, Gua Sha intervention was started on the sixth day after modeling in the Gua Sha group, Gua Sha + DMOG group, and Gua Sha + YC-1 group. The Gua Sha method referred to Yang's research [23]. Prior to Gua Sha, the dorsal fur of rats was shaved. The rat's head was gently secured with one hand to minimize visual stress, and the Gua Sha procedure was initiated only after the rat reached a calm state. The scraping intensity was carefully controlled to prevent pain-induced struggling during the operation. The Gua Sha board was used to scrape from top to bottom along the midline and both sides of the back, scraping each part 10–20 times until the skin was red, red granular, purplish–red, or dark red ecchymosis. Each rat was scraped for approximately 5 min once every other day, three times constituting one course of treatment, for a total of three courses. The Gua Sha + DMOG group and Gua Sha + YC-1 group were administered DMOG (60 mg/kg) and YC-1 (10 mg/kg), respectively, by intraperitoneal injection before each Gua Sha session. The sham surgery group and model group were fed normally and observed daily. The sham Gua Sha group received interventions at identical time points as the Gua Sha group but only underwent an immobilization procedure without any scraping manipulation. Each intervention was implemented in a fixed sequence. At the conclusion of the entire experimental process, isoflurane inhalation anesthesia was administered to the rats (flow rate: 2–3 L/min; concentration: 2.5%–3%). Euthanasia was then performed either through abdominal aortic puncture for blood withdrawal or via cardiac perfusion. Subsequently, L4 and L5 DRG tissues were removed. Throughout the procedure, every effort was made to minimize any potential suffering experienced by the rats. Assistants in the intervention stage will be informed of the grouping and intervention methods.

### 2.5. Behavioral Testing

**Figure 1:**
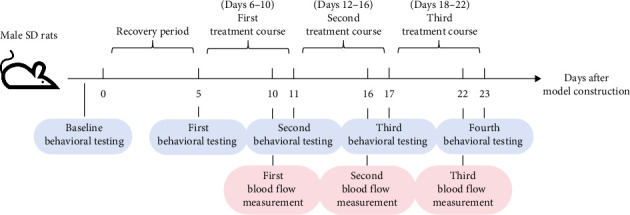
Experimental schedule.

Before surgery and on postoperative Days 5, 11, 17, and 23, the paw withdrawal threshold (PWT) and PWL of each group of rats were tested. During the PWT testing, the rats were placed individually in transparent plexiglass chambers (20 × 20 × 25 cm) on an elevated wire mesh platform. After 3 min habituation, mechanical sensitivity was measured using a Von Frey electronic algometer (Electronic Von Frey Anesthesiometer, IITC, United States of America) with a 0.4 mm diameter probe. The probe was applied vertically to the plantar surface of the right hind paw through the mesh, with force increasing from 0 to 50 g at a rate of 2 g/s. A positive withdrawal response was defined as either (1) a sudden paw retraction accompanied by licking/shaking, or (2) sustained elevation (> 2 s) of the stimulated limb. Three measurements were taken at 5-min intervals, and the mean value was recorded as PWT. The PWL of the rats was evaluated via a YLS-6B intelligent hot plate apparatus (Beijing Zhishuduobao, China) calibrated daily to 52.0 ± 0.2°C. Rats were restrained in a plexiglass cylinder (diameter 18 cm) allowing the right hind paw to contact the plate surface. The latency to withdraw or lick the paw was automatically recorded by an infrared sensor, with a 20 s cutoff to prevent tissue damage. Three trials were conducted at 10-min intervals, and the average latency was calculated (Figure 1).

### 2.6. Laser Doppler Flowmeter Detection of Blood Flow Perfusion

On the 10th, 16th, and 22nd days after surgery, that is, the last time of each course of Gua Sha, the blood flow perfusion of the rat's back before and after Gua Sha was recorded via a laser speckle blood flow imaging instrument (PeriCam PSI NR, Perimed, Sweden) (Figure 1). To ensure the rats remained still during the procedure, the rats were gently restrained by hand, similar to the method used during Gua Sha, with one hand lightly holding the head to prevent struggling. In a room temperature, awake, and quiet state, the scanning lens was aimed at the rat's back (distance 18 cm), and the average blood flow perfusion was calculated via PIMSoft blood flow meter imaging processing software after the detection value stabilized.

### 2.7. HE Staining and Immunofluorescence

At the end of all treatment courses, rats were injected with Hypoxyprobe-1 (60 mg/kg) and perfused to remove L4 and L5 DRGs. These were fixed, sectioned, and stained with HE for microscopic observation. For immunofluorescence, sections were antigen-retrieved, blocked, and incubated with FITC-MAb1 (1:100) as the primary antibody and rabbit anti-FITC was used as the secondary antibody before DAPI staining and observation under a fluorescence microscope.

### 2.8. Western Blot Analysis

Four L4 and L5 DRG tissues were randomly selected from the previously harvested specimens for western blot for each experimental group. DRG samples were homogenized in RIPA buffer, lysed, and centrifuged to obtain the supernatant. Protein concentration was measured, and equal amounts of protein (30 μg) were electrophoresed and transferred to a PVDF membrane. Membranes were blocked and incubated with *β*-actin (1:50,000), HIF-1*α* (1:3000), GLUT (1:500), PDK1 (1:2500), and PFKM (1:2500) antibodies at 4°C overnight, followed by incubation with goat anti-mouse IgG (1:5000) and goat anti-rabbit IgG (1:5000). ECL reagent was used for the development, and band intensities were quantified using Image*J* software, normalized to *β*-actin. Stripping buffer was used between developments to prevent band overlap.

### 2.9. Real-Time Quantitative PCR

Four L4 and L5 DRG tissues were randomly selected from the previously harvested specimens for PCR for each experimental group. Total RNA was extracted from DRG tissue according to the instructions of the FastPure Cell/Tissue Total RNA Isolation Kit. The concentration of total RNA was measured via a supermicro UV spectrophotometer. Reverse transcription was performed according to the instructions of the All-in-one RT Mix for qPCR to obtain cDNA. qPCR SYBR Green Master Mix (High ROX) was used to configure the premix for the qPCR experiments. The ΔΔCT relative quantification method was used to analyze the data. The sequences of the primer pairs are shown in Table 1.

### 2.10. LA and ATP Detection

Four L4 and L5 DRG tissues were randomly selected from the previously harvested specimens for LA and ATP detection for each experimental group. LA and ATP in DRG tissue were extracted via a LA content assay kit and an ATP assay kit according to the instructions. The LA detection results were measured at 570 nm via a Synergy H1MD Multimode Reader from BioTek, United States of America, which determines the absorbance values. For ATP detection, the luminescence intensity of each well was measured via the luminometer mode of the same multifunction plate reader.

### 2.11. ELISA Detection of Inflammatory Factors

Four L4 and L5 DRG tissues and blood samples were randomly selected from the previously harvested specimens for ELISA detection for each experimental group. Blood samples were centrifuged at 4°C at 3000 rpm for 15 min to separate the serum. The concentrations of IL-1β, TNF-*α*, and NF-κB in DRGs and serum were detected via rat IL-1β, TNF-*α*, and NF-κB ELISA kits following the manufacturer's instructions, and the absorbance at 450 nm was measured via the same multifunction plate reader.

### 2.12. Statistical Analysis

GraphPad Prism 8.0.1 software was used for graphing and statistical analysis. The experimental data are expressed as mean ± SDs. The western blot, qPCR, ELISA, immunofluorescence, LA, and ATP detection results were analyzed via one-way ANOVA. The blood flow changes before and after Gua Sha, PWT, and PWL detection were analyzed via two-way ANOVA. Multiple comparisons of blood flow changes before and after Gua Sha data were performed via the Sidak's multiple comparisons test, and multiple comparisons of PWT and PWL data were performed via Tukey's multiple comparisons test, with *p* < 0.05 as the standard for statistically significant differences.

## 3. Results

### 3.1. Gua Sha Promotes Blood Flow Perfusion in the Backs of LDH Rats and Improves DRG Tissue Hypoxia

**Figure 2:**
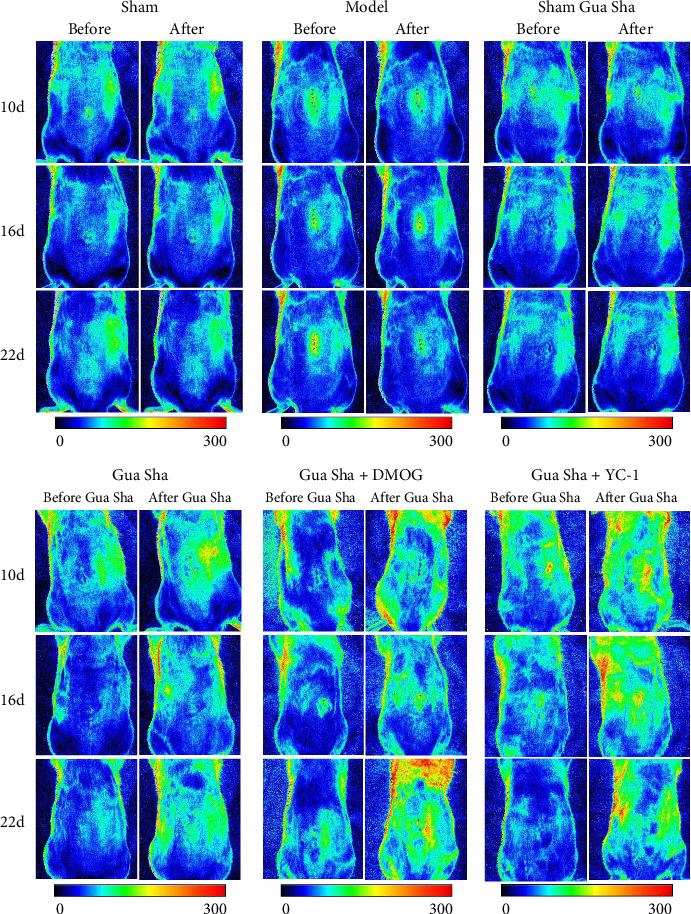
Representative laser imaging images of blood flow perfusion before and after Gua Sha.

**Figure 3:**
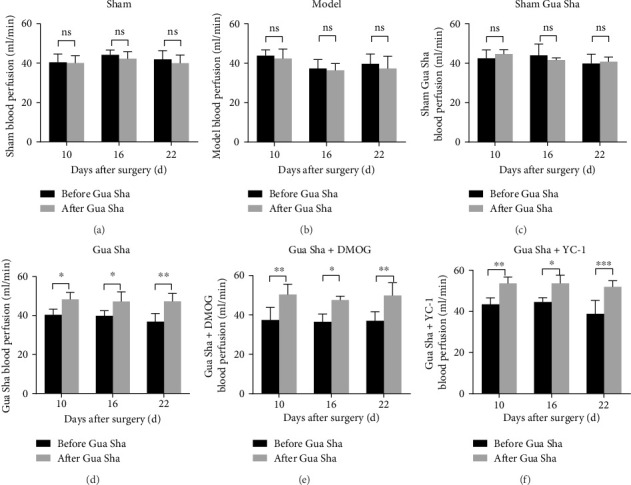
Effects of Gua Sha on blood flow perfusion in the backs of rats. (a–f) Quantitative data of blood flow perfusion before and after Gua Sha in each group of rats. The values are expressed as mean ± SDs. ⁣^∗^*p* < 0.05, ⁣^∗∗^*p* < 0.01, and ⁣^∗∗∗^*p* < 0.001; ns, no significance; *n* = 4.

**Figure 4:**
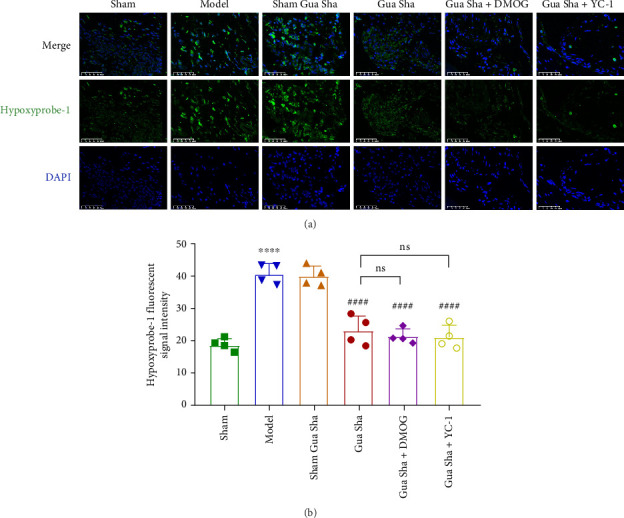
Effects of Gua Sha on hypoxia in DRG tissue. (a) Representative IF images of Hypoxyprobe-1 and DAPI in DRG tissue from each group of rats. (b) Image *J* quantitative analysis of Hypoxyprobe-1 fluorescence signal intensity in each group. The values are expressed as mean ± SDs. ⁣^∗∗∗∗^*p* < 0.0001 vs. the sham group; ^####^*p* < 0.0001 vs. the model group; ns, no significance; *n* = 4.

The results revealed that the blood flow perfusion in the backs of the rats in the Gua Sha, Gua Sha + DMOG, and Gua Sha + YC-1 groups significantly increased after Gua Sha treatment compared to before treatment. In contrast, there was no significant change in blood flow perfusion in the backs of the rats that did not undergo Gua Sha. There was no significant difference in the rats in each group before the treatment at any time point. At the same time, after the treatment, there was no difference between the model, sham group, and the sham Gua Sha group, and there was no difference between the Gua Sha, Gua Sha + DMOG group, and the Gua Sha + YC-1 group. However, the blood flow perfusion on the back of the rats that underwent Gua Sha treatment was significantly higher than that of the rats that did not undergo Gua Sha treatment or had a tendency to increase (Figures 2 and 3). To observe the effect of Gua Sha on hypoxia in DRG tissue, we administered Hypoxyprobe-1 intraperitoneally to the rats. Hypoxyprobe-1 binds to proteins within cells with oxygen concentrations ≤ 14 μM, forming a complex. This implies that the stronger the fluorescence is, the more severe the hypoxia. Immunofluorescence detection revealed that the DRG tissue of the model group rats was significantly hypoxic compared to that of the sham group rats. Compared to that in the model group, the hypoxia in DRG tissue in the Gua Sha, Gua Sha + DMOG, and Gua Sha + YC-1 groups was significantly greater, with no significant differences between the Gua Sha group and the Gua Sha + DMOG and Gua Sha + YC-1 groups (Figure 4). These findings indicate that Gua Sha promotes local blood flow perfusion and can improve hypoxia in DRG tissue. Notably, the promotion of blood flow perfusion and improvement in DRG tissue hypoxia by Gua Sha were not affected by DMOG or YC-1.

### 3.2. Gua Sha Regulates Metabolic Reprogramming and Metabolites by Inhibiting HIF-1α/Glycolysis

**Figure 5:**
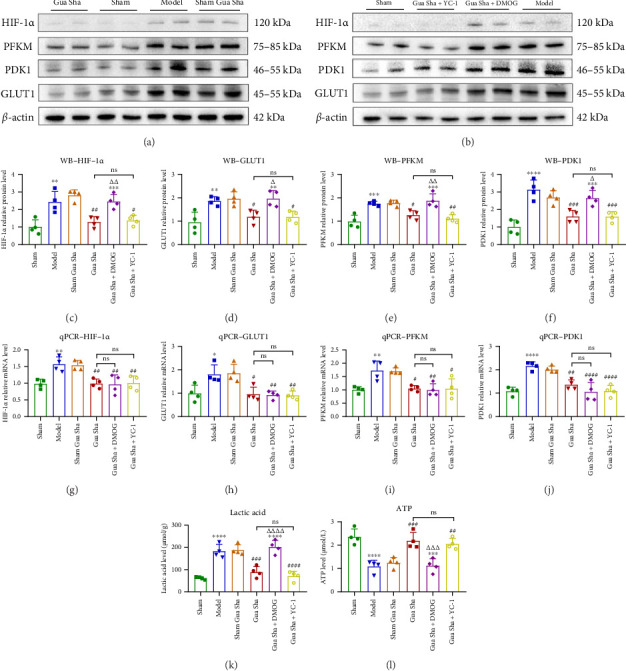
Gua Sha inhibits HIF-1α and its downstream factors and regulates glycolytic pathways, controlling metabolic reprogramming and metabolites. (a–b) Representative images of protein imprinting, the samples derived from the same experiment, and gels/blots were processed in parallel. (c–f) Relative protein expression levels of HIF-1α, GLUT1, PFKM, and PDK1 in DRG tissue; the data were normalized to those of the respective sham groups. (g–j) Relative mRNA expression levels of HIF-1α, GLUT1, PFKM, and PDK1 in DRG tissue. (k) Lactic acid levels in DRG tissue. (l) ATP levels in DRG tissue. The values are expressed as mean ± SDs. ⁣^∗^*p* < 0.05, ⁣^∗∗^*p* < 0.01, ⁣^∗∗∗^*p* < 0.001, and ⁣^∗∗∗∗^*p* < 0.0001 vs. the sham group; ^#^*p* < 0.05, ^##^*p* < 0.01^###^*p* < 0.001, and ^####^*p* < 0.0001 vs. the model group; ^△^*p* < 0.05, ^△△^*p* < 0.01, ^△△△^*p* < 0.001, and ^△△△△^*p* < 0.0001 vs. the Gua Sha group; ns, no significance; *n* = 4.

Previous studies have confirmed that Gua Sha can improve hypoxia in DRG tissue. To observe whether the oxygen regulatory factor HIF-1α and metabolic reprogramming are also regulated by Gua Sha, we detected changes in the protein and mRNA levels of HIF-1α and its downstream factors. We also measured the levels of metabolic products, LA, and ATP. The western blot results revealed that Gua Sha reduced the protein expression levels of HIF-1α, GLUT1, PDK1, and PFKM, which were significantly increased in the model group. In the Gua Sha + DMOG group, the levels of these indicators were significantly greater than those in the Gua Sha group, whereas there were no significant differences between the Gua Sha + YC-1 group and the Gua Sha group (Figures 5(a), 5(b), 5(c), 5(d), 5(e), and 5(f)). The qRT-PCR results revealed that Gua Sha reduced the mRNA expression levels of HIF-1α, GLUT1, PDK1, and PFKM, which were significantly increased in the model group. Notably, there were no significant differences in these indicators between the Gua Sha group and the Gua Sha + DMOG and Gua Sha + YC-1 groups (Figures 5g, 5h, 5i, and 5j). These results indicate that Gua Sha inhibits glycolytic flux in DRG tissue by suppressing HIF-1*α*. We further detected the metabolic products LA and ATP. LA level detection revealed that the LA level in DRG tissue was significantly greater in the model group than in the sham group and was significantly lower in the Gua Sha group than in the model group. Compared to the Gua Sha + DMOG group, the Gua Sha group presented a significant increase, whereas there was no significant difference between the Gua Sha + YC-1 group and the Gua Sha group (Figure 5(k)). ATP level detection revealed that the ATP content in the DRG tissue of the model group was significantly lower than that in the sham group and was significantly greater in the Gua Sha group than in the model group. Compared to the Gua Sha + DMOG group, the Gua Sha group presented a significant decrease, while there was no significant difference between the Gua Sha + YC-1 group and the Gua Sha group (Figure 5(l)). These results indicate that LDH leads to hypoxic DRG tissue, causing an increase in HIF-1α and inducing metabolic reprogramming, with increased glycolysis. Gua Sha can improve hypoxia in the DRG and suppress HIF-1α and its downstream factors, reducing LA while promoting ATP production.

### 3.3. Gua Sha Alleviates RIP by Inhibiting HIF-1α–Mediated Metabolic Reprogramming

**Figure 6:**
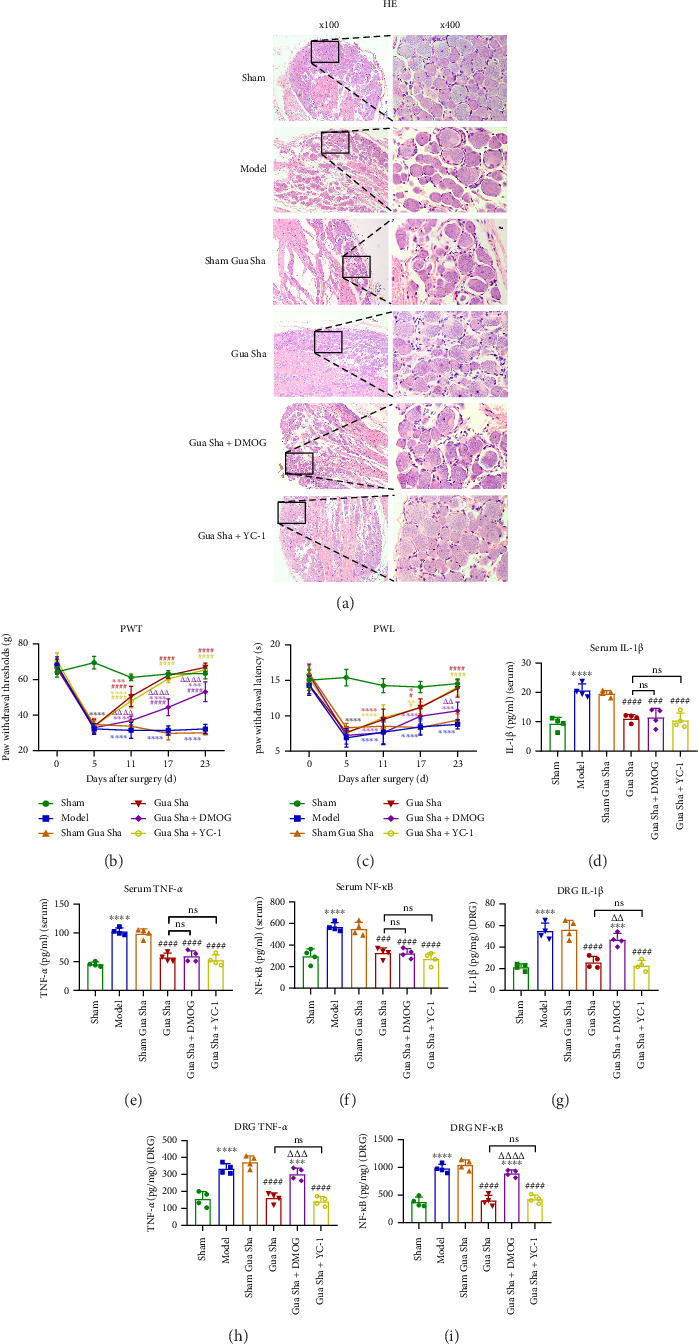
Effects of Gua Sha on DRG tissue, pain, and inflammation. (a) Representative images of HE–stained rat DRG tissue. (b) Changes in PWT. (c) Changes in PWL. (d–f) ELISA results showing the levels of IL-1β, TNF-*α*, and NF-κB in the serum. (g–i) ELISA detection of IL-1β, TNF-*α*, and NF-κB in DRG tissue. The values are expressed as mean ± SDs. ⁣^∗^*p* < 0.05, ⁣^∗∗^*p* < 0.01, ⁣^∗∗∗^*p* < 0.001, and ⁣^∗∗∗∗^*p* < 0.0001 vs. the sham group; ^#^*p* < 0.05, ^###^*p* < 0.001, and ^####^*p* < 0.0001 vs. the model group; ^△△^*p* < 0.01, ^△△△^*p* < 0.001, and ^△△△△^*p* < 0.0001 vs. the Gua Sha group; ns, no significance; *n* = 4.

HE staining revealed that the pseudounipolar neuronal cells in the DRG tissue of the sham group rats were intact in morphology, clear in boundary, orderly in arrangement, and had distinct cell nuclei. In contrast, the pseudounipolar neuronal cells in the model group, sham Gua Sha group, and Gua Sha + DMOG group were disordered in morphology, had vague boundaries, were loosely arranged, and some swollen cells with unclear nuclear staining. Compared to those in the model group, the morphology of pseudounipolar neuronal cells in the Gua Sha and Gua Sha + YC-1 groups improved, with clear boundaries, reduced cell looseness and swelling, and distinct cell nuclei (Figure 6a). The ELISA results revealed that the levels of IL-1β, TNF-*α*, and NF-κB in the serum and DRG of the Gua Sha group were significantly lower than those in the model group. Compared to the Gua Sha + YC-1 group, the Gua Sha-1 group presented no significant differences in these indicators. Interestingly, the levels of IL-1β, TNF-*α*, and NF-κB in the DRG tissue in the Gua Sha + DMOG group were significantly greater than those in the Gua Sha group, whereas there was no difference in the serum IL-1β, TNF-*α*, and NF-κB levels between the Gua Sha + DMOG and Gua Sha groups (Figures 6(g), 6(e), 6(f), 6(g), 6(h), and 6(i)). On the fifth day after surgery, the PWT and PWL of the rats in all the groups except those in the sham group significantly decreased. On the 11th day after surgery, the PWT of the Gua Sha group was significantly greater than that of the model group. On the 17th day after surgery, the PWL of the Gua Sha group was significantly greater than that of the model group. The curve of the Gua Sha + YC-1 group was highly consistent with that of the Gua Sha group, with no significant difference at any time point. Notably, although the PWT and PWL of the Gua Sha + DMOG group on the 23rd day were still significantly lower than those of the Gua Sha group, they were still significantly greater than those of the model group (Figures 6(b) and 6(c)). These results indicate that by inhibiting HIF-1α, Gua Sha can effectively alleviate the inflammatory response and pain symptoms in LDH rats and reduce DRG damage.

## 4. Discussion

The inflammatory response is the foundation of RIP, and inflammation can lead to tissue hypoxia. Hypoxia can further exacerbate inflammation and enhance glycolysis through HIF-1α–mediated metabolic reprogramming, resulting in an acidic environment [4, 14–17]. Recent *in vitro* studies have also revealed that hypoxic and acidic environments can sensitize DRG neurons [5]. Studies have shown that hypoxic and acidic environments are characteristics of degenerative IVDs [25–29]. These studies all illustrate the link between hypoxia and RIP, but there is a lack of corresponding *in vivo* studies exploring the response of DRG tissue. In this study, LDH rats presented a decreased PWT, a shortened PWL, increased levels of inflammatory factors in the serum and DRG, and damage to DRG tissue. Notably, we observed hypoxia and increased LA levels in DRG tissue. After Gua Sha intervention, these indicators returned to normal levels, and we also observed enhanced microcirculation perfusion in the back. These findings indicate that LDH indeed creates hypoxic and acidic environments and is involved in the development of RIP *in vivo*. Gua Sha alleviated pain and inflammatory responses in LDH rats and ameliorated DRG tissue damage. It also reduces the LA concentration by promoting microcirculation, thereby improving DRG sensitization and inflammatory responses.

As an oxygen regulatory factor that is stably expressed under hypoxic conditions, HIF-1α is expressed in IVDs and nucleus pulposus and is involved in the process of IVD degeneration [11, 12, 25]. Although recent studies have shown that elevated HIF-1α can delay IVD degeneration and has a protective effect on neurons in *in vitro* ischemia models [30–32], previous studies have shown that HIF-1α and its mediated metabolic reprogramming can regulate various inflammatory cell phenotypes, including those of macrophages and T cells, and promote the expression of inflammatory genes [33, 34]. Therefore, the specific impact of HIF-1α on DRG tissue *in vivo* remains unknown. In our study, the protein and mRNA expression of HIF-1α in the DRG tissue of LDH rats was abnormally increased. This observation suggests that HIF-1α is likely involved in the progression of RIP and may serve as a potential target for Gua Sha intervention in the treatment of RIP.

To observe whether HIF-1α caused metabolic reprogramming within DRG tissue, we detected the protein and mRNA expression of GLUT1, PDK1, and PFKM. GLUT1 is involved in glucose absorption. PDK1 inhibits the activity of pyruvate dehydrogenase by phosphorylation, thereby inhibiting the citric acid cycle and increasing LA production. PFKM is an isozyme of PFK1 and is also a rate-limiting enzyme in glycolysis. They play a key role in the glycolytic process and are regulated by HIF-1α [35–37]. Our results revealed that the protein and mRNA expression of GLUT1, PDK1, and PFKM in the DRG tissue of LDH rats significantly increased, indicating an increase in glycolytic flux. Compared to OXPHOS, glycolysis is less efficient at producing ATP; thus, metabolic reprogramming maintains ATP production by increasing glycolytic flux while producing a large amount of LA [38]. Together, the decrease in ATP and increase in LA in the DRG tissue of LDH rats support the enhancement of glycolytic pathways. Notably, Gua Sha reduced the protein and mRNA expression of GLUT1, PDK1, and PFKM and increased ATP while decreasing LA content in DRG tissue. These results indicate that when RIP occurs, HIF-1α–mediated metabolic reprogramming occurs within DRG tissue, leading to the production of large amounts of LA and the creation of an acidic environment, further causing DRG sensitization. A hypoxic environment further exacerbates sensitivity. Gua Sha has the ability to improve hypoxia, inhibit HIF-1α–mediated metabolic reprogramming, and reduce LA.

To verify whether Gua Sha alleviates RIP by regulating HIF-1α–mediated metabolic reprogramming, we chose DMOG as a HIF-1α agonist and YC-1 as a HIF-1α inhibitor. DMOG is a prolyl hydroxylase (PHD) inhibitor. PHD degrades HIF-1α through hydroxylation, and DMOG can inhibit this process and stabilize HIF-1α [39]. YC-1 was initially developed as a potential treatment for circulatory disorders because of its inhibitory effects on platelet aggregation and vascular contraction [40]. With the discovery of the inhibitory effect of YC-1 on HIF-1α, it has been widely applied in the field of antitumor research [41]. Previous studies have shown that DMOG and YC-1 regulate only the protein level of HIF-1α and do not affect its mRNA level, which is consistent with our research results [32, 42, 43]. Both DMOG and YC-1 exert their effects on HIF-1α at the protein level. DMOG stabilizes HIF-1α protein by inhibiting PHD, while YC-1 promotes the binding of HIF-1α to pVHL, accelerating its ubiquitination and degradation. This, in turn, further affects downstream proteins of HIF-1α, with no significant impact on mRNA levels. We observed that after Gua Sha, the expression of the HIF-1α, GLUT1, PDK1, and PFKM proteins, which should have decreased, increased in response to the addition of DMOG. Moreover, LA levels increased, and ATP levels decreased. The agonistic effect of DMOG on HIF-1α also caused a decrease in the PWL and PWT and an increase in the expression of inflammatory factors in the DRG. However, inhibiting HIF-1α did not significantly affect the experimental results. Notably, the addition of DMOG had no significant effect on the levels of inflammatory factors in the serum. After the addition of DMOG, although the effect was not as good as that of the Gua Sha group, the PWT was still partially improved. This indicates that Gua Sha has the effect of alleviating systemic inflammation, which is consistent with the results of our previous studies [23]. However, the addition of DMOG had no effect on serum inflammatory factors, which may suggest that Gua Sha still exerts its anti-inflammatory effects through other mechanisms. At the same time, the partial improvement of PWL after the addition of DMOG suggests that Gua Sha may also alleviate RIP through other pathways. In summary, through verification experiments, we have proven that HIF-1α–mediated metabolic reprogramming is involved in the progression of RIP. One of the mechanisms by which Gua Sha alleviates RIP involves the inhibition of HIF-1α–mediated metabolic reprogramming.

The relationships between Gua Sha, RIP, and glycolysis remain controversial. Previous studies have shown that Gua Sha promotes the GLUT4/glycolytic pathway in muscle tissue to promote muscle regeneration [44]. We speculate that the differences may be due to the different tissues tested, which may indirectly explain the complex regulatory mechanism of Gua Sha throughout the body. Therefore, it is still necessary to further explore the effect mechanism of Gua Sha in alleviating RIP. Some studies also suggest that enhancing glycolytic metabolism is essential for protecting DRG neurons from hypoxia-induced mitochondrial damage [45]. However, similar to our study, some studies have reported that inhibiting glycolysis can alleviate neuropathic pain in the DRG [46]. Future research is needed to further explore the relationship between metabolism and RIP. Notably, considering that YC-1 was originally used to treat circulatory disorders, it has a similar therapeutic mechanism to Gua Sha's promotion of microcirculation blood flow. These findings suggest that treatments aimed mainly at improving microcirculation may also be used to alleviate RIP. Future studies can observe the alleviating effect of microcirculation improvement on RIP.

In addition, this study has several limitations. Due to the use of whole DRG tissue in our study methods, we were unable to investigate which specific cell type is involved in the HIF-1α–mediated metabolic reprogramming. However, previous studies have shown that HIF-1α is involved in the excessive activation of satellite glial cells (SGCs) in DRG tissue, and the activated SGCs in turn induce the release of IL-1β and TNF-*α* [47, 48]. In light of the decreased levels of IL-1β and TNF-*α* in DRG tissue following Gua Sha in our study, we speculate that Gua Sha may exert its effects by influencing SGCs. This highlights the potential link between Gua Sha, HIF-1*α*, and SGCs, which warrants further investigation through cell-specific targeting studies in the future. Owing to the particularity of Gua Sha's manipulation, we were unable to conduct *in vitro* experiments. Moreover, we inhibited and stimulated only HIF-1*α* and did not further verify the impact of factors and metabolic products related to metabolic reprogramming on RIP. This may limit our comprehensive understanding of the mechanism by which Gua Sha alleviates RIP, and future research can further explore these mechanisms.

## 5. Conclusion

**Figure 7:**
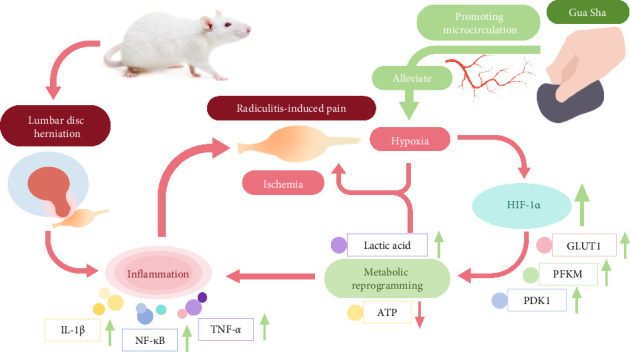
Schematic illustration of the mechanism of Gua Sha improving RIP through HIF-1α–mediated metabolic reprogramming.

In summary, this study demonstrated that Gua Sha can alleviate RIP by promoting microcirculation blood flow perfusion and relieving hypoxia in DRG tissue. It inhibits the enhancement of glycolytic pathways caused by HIF-1α–mediated metabolic reprogramming, reduces LA produced by glycolysis, prevents DRG sensitization caused by acidic stimulation, and reduces inflammatory responses (Figure 7). This study provides new insights into the molecular mechanisms by which Gua Sha treats RIP and LDH. Further studies are needed to reveal the detailed mechanisms by which Gua Sha alleviates RIP by inhibiting metabolic reprogramming and other potential pathways.

Gua Sha alleviates hypoxia in DRG tissue caused by inflammation and inhibits HIF-1*α* and its downstream targets GLUT1, PFKM, and PDK1, thereby inducing metabolic reprogramming. This process subsequently suppresses glycolysis, reduces lactate production, mitigates the sensitization of DRG, and inhibits the release of inflammatory factors IL-1β, TNF-*α*, and NF-κB, thus alleviating RIP.

## Figures and Tables

**Table 1:** Primer pair sequences.

Gene	Gene ID	Primer	Sequence 5′-3′
*β*-actin	81822	Forward	GGAGATTACTGCCCTGGCTCCTA
		Reverse	GACTCATCGTACTCCTGCTTGCTG

HIF-1α	29560	Forward	GCGGCGAGAACGAGAAGAA
		Reverse	GGAAGTGGCAACTGATGAGCAA

PFKM	65152	Forward	GGCGGAGGAGAGCTAAAACTA
		Reverse	GACCGCAGCATTCATACCTTG

PDK1	116551	Forward	CCATATCACGCCTCTATGCAC
		Reverse	TCTTTCGATGGACTCCGTTG

GLUT1	24778	Forward	ACTGTGGTGTCGCTGTTCG
		Reverse	GGCCACGATACTCAGATAGGAC

## Data Availability

The data that support the findings of this study are available from the corresponding author upon reasonable request.

## Supporting Information

S1. ARRIVE author checklist-full.
